# Cognitive Functioning in Turner Syndrome: Addressing Deficits Through Academic Accommodation

**DOI:** 10.1089/whr.2019.0019

**Published:** 2020-05-27

**Authors:** Gabrielle E. Reimann, Leora E. Comis, Martha M. Bernad Perman

**Affiliations:** Rehabilitation Medicine Department, National Institutes of Health, Bethesda, Maryland, USA.

**Keywords:** accommodations, educational attainment, cognition, support services

## Abstract

***Background:*** The cognitive profile of Turner syndrome, a genetic disorder resulting from partial or complete X-chromosome deletion, presents characteristic deficits. Despite this, studies have yet to evaluate how deficits translate into and are compensated for in academic settings. This study seeks to explore cognitive functioning, as well as the accessibility and development of academic accommodations in females with Turner syndrome from adolescence to adulthood.

***Materials and Methods:*** This cross-sectional study took place at the National Institutes of Health. Females with Turner syndrome (age range: 10–68; *n* = 142) were evaluated on need for and procurement of academic accommodations. Cognitive functioning was evaluated in participants aged 20 years and older (*n* = 101), as per the age validation of the Repeatable Battery for the Assessment of Neuropsychological Status. Data were analyzed using descriptive statistics, one-sample comparisons, and analyses of variance.

***Results:*** Females with Turner syndrome scored significantly lower than the normative population on visuospatial (*p* < 0.001), delayed memory (*p* < 0.001), and overall (*p* < 0.001) functioning. About 25.9% of participants reported that accommodations were not needed, despite displaying one or more cognitive deficits. Approximately 12.7% reported needing but not receiving accommodations, however, this is only reported by females 30 years and older; no females aged 10–29 years indicated this discrepancy.

***Conclusions:*** Findings suggest that procurement of academic accommodations has increased within recent decades. Still, there is a discrepancy between those displaying cognitive deficits and those receiving academic accommodations. We highlight frequently received accommodations so that students and professionals can target deficits with appropriate accommodations.

## Introduction

Turner syndrome is a common genetic disorder, occurring in ∼1 in 2000–2500 live female births.^1,2^ It is characterized by partial or complete deletion of one X-chromosome, and is often associated with short stature, ovarian failure, dysmorphia, hearing loss, cardiac complications, and cognitive deficits.^3^

Studies on this disorder have advanced understanding of how X-chromosome deletion affects cognitive development. Previous findings report a characteristic cognitive profile in females with Turner syndrome.^4,5^ Overall intellectual functioning appears to be intact, with average or above average intelligence present in 90% of the population.^6^ Females with Turner syndrome also display preserved verbal ability, with average to above average performance on vocabulary development and reading abilities.^5,7–9^ Although many functions are intact, findings also report specific cognitive deficits that may impede academic achievement. The chromosomal aneuploidy has implications for deficits involving numerical processing, suggesting an X-linked component to arithmetic abilities.^10^ As a hallmark impairment among the Turner syndrome population, visuospatial deficits often translate to difficulty in mathematics.^5,11^ Studies also describe recall deficits, particularly in word retrieval or sentence completion, which may impair academic activities such as group discussions or oral presentations.^5,9^ Furthermore, findings highlight attentional deficits, with attention deficit hyperactivity disorder diagnoses displayed in 24% of the Turner syndrome population.^12^

Recent decades have pushed for and enacted federal laws entitling students with learning disabilities to receive academic accommodations. Individuals with Disabilities Education Act, which addresses the special education needs of students, made way for children with disabilities to be entitled to a free and appropriate public education.^13^ Furthermore, Americans with Disabilities Act enables changes in academic environments to provide equal opportunity to individuals with disabilities.^14^

In light of these laws, researchers have explored resulting accessibility, development, and implementation of accommodations for individuals with disabilities. Studies are dedicated to informing teachers and other professionals on best practices to support educational attainment for students with these disorders.^15,16^ Accommodations research often focuses on common and well-known disorders, such as autism spectrum disorder.^15–17^ Although Turner syndrome is a common genetic disorder displaying clear cognitive deficits, it may not be as well known as other disorders. As a result, research regarding Turner syndrome-related academic accommodations is understated.

Although previous studies have explored cognitive anomalies linked to X-monosomy, researchers have yet to evaluate how these cognitive deficits translate into and are compensated for in an academic setting. In the absence of such knowledge, the development of effective intervention strategies to help accommodate for these deficits will likely remain difficult. This study explores cognitive deficits and provision of academic accommodations in females with Turner syndrome. We seek to evaluate the history and development of academic accommodations in a sample of females with Turner syndrome in adolescence to late adulthood. By examining participants varying in age, we aim to investigate the progress of accommodation procurement over recent decades.

## Materials and Methods

### Procedure

Through the *Eunice Kennedy Shiver* National Institute of Child Health and Human Development (NICHD), females with Turner syndrome were recruited for a multidisciplinary study, with the purpose of examining the clinical and genetic underpinnings of Turner syndrome. The research protocol was approved by the NICHD Institutional Review Board (NICHD; 2000-CH-0219). The current cross-sectional study was conducted as a subpart of this protocol, assessing cognitive functioning and academic accommodations through cognitive assessment and vocational interviews.

This study took place at the National Institutes of Health (NIH) Clinical Center in Bethesda, MD, between December 2008 and June 2012. Participants or their legal guardians granted informed consent to engage in medical and neurocognitive testing over a 5-day inpatient stay. Researchers of this study conducted a semistructured interview to collect participants' demographic information and academic accommodation history. Parents or guardians of minors also verified accommodation history. A certified rehabilitation counselor administered the Repeatable Battery for the Assessment of Neuropsychological Status (RBANS).

### Participants

All participants were recruited through ClinicalTrials.gov online postings, physician referrals, Turner syndrome advocacy groups, and medical journal advertisements. Enrollment eligibility under this protocol was contingent on the following criteria: (1) evidence of an X-chromosomal abnormality (or at least 80% 45X lymphocytes for those with a 45X/46XX karyotype), (2) at least 10 years of age, (3) nonpregnant, (4) no coexisting autosomal defects, and (5) individual consent or parental assent for minors. Participant karyotype was verified through blood sampling taken at the initial screening.

Of the participants (*n* = 160) enrolled in this study, 18 were excluded due to non-Turner syndrome karyotype abnormalities (*n* = 4), incomplete testing portfolios due to scheduling conflicts (*n* = 13), or severe intellectual disability (*n* = 1). The final accommodation analysis included 142 participants (Table 1). Cognitive deficit analyses were based on an RBANS version validated on individuals 20 years and older (Table 2). Therefore, participants aged 10–19 years (*n* = 41) were not included in analysis of RBANS, yielding a sample size of 101 participants.^18^

**Table 1.** Demographic Characteristics, *n* (%), of Turner Syndrome Sample at Time of Evaluation

Characteristics	10–19 (n = 41)	20–29 (n = 19)	30–49 (n = 59)	50–69 (n = 23)	Total (n = 142)
Race					
Caucasian	35 (85.4)	16 (84.2)	51 (86.4)	20 (87.0)	122 (85.9)
African American	1 (2.4)	0	4 (6.8)	3 (13.0)	8 (5.6)
Hispanic	2 (4.9)	2 (10.5)	2 (3.4)	0	6 (4.2)
Other^a^	3 (7.3)	1 (5.3)	2 (3.4)	0	6 (4.2)
Region of the United States					
Northeast	6 (14.6)	2 (10.5)	7 (11.9)	5 (21.7)	20 (14.1)
Midwest	9 (22.0)	4 (21.1)	11 (18.6)	7 (30.4)	31 (21.8)
South	14 (34.1)	8 (42.1)	25 (42.4)	6 (26.1)	53 (37.3)
West	10 (24.4)	4 (21.1)	13 (22.0)	5 (21.7)	32 (22.5)
International	2 (4.9)	1 (5.3)	3 (5.1)	0	6 (4.2)
Education					
Current student	33 (80.5)	4 (21.1)	0	0	37 (26.1)
High school, GED or less	2 (4.9)	5 (26.3)	12 (20.3)	3 (13.0)	22 (15.5)
Some college	1 (2.4)	1 (5.3)	17 (28.8)	6 (26.1)	25 (17.6)
College/postgraduate	0	9 (47.4)	30 (50.8)	14 (60.9)	53 (37.3)
Unreported	5 (12.2)	0	0	0	5 (3.5)
Employment status					
Part-time	1 (2.4)	6 (31.6)	7 (11.9)	7 (30.4)	21 (14.8)
Full-time	0	6 (31.6)	43 (72.9)	10 (43.5)	59 (41.5)
Unemployed	0	2 (10.5)	6 (10.2)	0	8 (5.6)
Other^b^	0	1 (5.3)	3 (5.1)	6 (26.1)	10 (7.0)
Current student	32 (78.0)	4 (21.1)	0	0	36 (25.4)
Part-time work/current student	2 (4.9)	0	0	0	2 (1.4)
Unreported	6 (14.6)	0	0	0	6 (4.2)
Karyotype					
45X	24 (58.5)	17 (89.5)	41 (69.5)	16 (69.6)	98 (69.0)
Other	17 (41.5)	2 (10.5)	18 (30.5)	7 (30.4)	44 (31.0)

^a^ Race (Asian, Middle Eastern Pacific Islander, biracial).

^b^ Employment status (retired, unable to work due to illness).

GED, general education development.

**Table 2.** Cognitive Deficits and Characteristics, *n* (%), by Age

	20–29 (n = 19)	30–49 (n = 59)	50–69 (n = 23)	Total (n = 101)
Cognitive deficits				
No deficits	10 (52.6)	34 (57.6)	18 (78.3)	62 (61.4)
One domain	6 (31.6)	17 (28.8)	5 (21.7)	28 (27.7)
Two domains	3 (15.8)	6 (10.2)	0	9 (8.9)
Three or more domains	0	2 (3.4)	0	2 (2.0)
Deficits shown				
Immediate	2 (10.5)	3 (5.1)	0	5 (5.0)
Visuospatial	8 (42.1)	20 (33.9)	2 (8.7)	30 (29.7)
Language	0	4 (6.8)	0	4 (4.0)
Attention	2 (10.5)	4 (6.8)	2 (8.7)	8 (7.9)
Delayed memory	0	6 (10.2)	1 (4.3)	7 (6.9)
Total score	0	6 (10.2)	0	6 (5.9)

This table reports only those with RBANS scores. Minors who were not administered the RBANS (*n* = 41) are not included.

RBANS, Repeatable Battery for the Assessment of Neuropsychological Status.

### Measures

Researchers collected data on participants' age at evaluation, education, race, height, and employment status. NIH Electronic Medical Record provided participants' region of residence at time of evaluation. Education was categorized as (1) current student, (2) high school (high school, high school equivalent, or less), (3) some college (some college, some Associate's degree, or completed Associate's degree), (4) college and postgraduate degree, and (5) unreported. Race was categorized as (1) Caucasian, (2) African American, (3) Hispanic, and (4) other (Asian, Middle Eastern, Pacific Islander, or biracial). Employment status was categorized as (1) full-time employment (35 or more hours per week), (2) part-time employment (<35 hours per week), (3) unemployed (unemployed by choice or unable to find work), (4) other (retired or unable to work due to illness), (5) current student, and (6) part-time employment and current student, and (7) unreported. Residential region of the United States was categorized as (1) Northeast, (2) Midwest, (3) South, (4) West, and (5) International.^19^ Karyotype was categorized as (1) 45X (complete deletion of one X-chromosome) and (2) other (mosaicism or partial deletion within the X-chromosome).

Researchers obtained participants' self-reported academic accommodations history, including current and past needed and received accommodations. Accommodation was defined in this study as modifications to tasks, environment, or procedures to equalize opportunity for individuals with disabilities to participate in academic settings.^20^

Accommodation history was categorized as (1) needed/received (accommodations needed and received), (2) not needed (accommodations not needed and not received), (3) needed/not received (accommodations needed but not received), and (4) unreported. Accommodations were categorized as (1) environmental accommodations (*e.g.*, seat placement, quiet testing space, ergonomic adjustments, wheelchair, and elevator access), (2) extra time (*e.g.*, additional time received during examinations or class assignments), (3) material resources (*e.g.*, use of calculators, extra notes, hearing aids, take-home materials, and recorders), (4) tutoring, and (5) remedial classes. Some individuals may have received and reported more than one type of accommodation.

RBANS is a brief test of neurocognitive functioning. This assessment uses 12 subtests to evaluate five specific cognitive domains (immediate memory, visuospatial/constructional, language, attention, and delayed memory), as well as overall functioning (total score). These six scores are age standardized to create index scores, scaled to have a mean of 100 and a standard deviation (SD) of 15.

RBANS uses the following ranges to classify scores: below average (79 and below), average (80–109), and high average to above average (110 and above). In this study, scores falling 1.5 SD below average were considered indicative of mild cognitive deficits. The normative sample was derived from individuals with no cognitive deficits, aged 20–89 years (*n* = 540).^18^ This version of the RBANS has two psychometrically equivalent forms: Forms A and B. In this study, researchers administered RBANS Form A.^18^ This study used the original version of RBANS released in 1998 as this was the most recent version of the assessment at the onset and through the majority of the study's duration.^18^

### Statistical analysis

We used descriptive statistics to evaluate demographic distributions and academic accommodation history. We used one-sample *t*-test to compare differences in RBANS scores between the Turner syndrome sample and the normative population, with alpha levels adjusted for multiple comparisons using the Bonferroni–Holm method.^21^ We used the Statistical Package for the Social Sciences (SPSS) 24 and R 3.5.0 to conduct these analyses (International Business Machines Corporation SPSS, Chicago, IL; R Foundation for Statistical Computing, Vienna, Austria).

## Results

Overall analyses included data from 142 participants (Table 1). Mean age at evaluation was 33.71 ± 15.8 (SD) years (range: 10–68 years), and mean height was ∼144.78 cm. Approximately 37% of this sample completed college or postgraduate education, 33.1% had completed less than a college education, and 26.1% were currently students.

Participants evaluated for cognitive deficits (*n* = 101) most frequently displayed visuospatial difficulty (29.7%; Table 2). Participants scored significantly lower than the normative population on visuospatial (*M* = 85.44, *p* < .001), delayed memory (*M* = 94.86, *p* < .001), and total score (*M* = 94.16, *p* < .001) index scores (Table 3). Turner syndrome immediate memory, language, and attention index scores were not significantly different than those of the normative population.

**Table 3.** Comparison Between Normative RBANS Scores Versus Turner Syndrome RBANS Index Scores (*n* = 101)

	Mean Turner syndrome score	p	Mean difference
Immediate memory	103.60	0.02	3.60
Visuospatial	85.44	<0.001^**^	−14.56
Language	97.16	0.017	−2.84
Attention	98.54	0.33	−1.45
Delayed memory	94.86	<0.001^**^	−5.13
Total score	94.16	<0.001^**^	−5.84

Normative RBANS scores: mean = 100, standard deviation = 15; significance of *p*-values was adjusted for multiple comparisons using Bonferroni–Holm method.

^**^ *p* < 0.001.

Academic accommodation history showed that 46.5% of participants self-reported not needing accommodations and 30.3% identified needing and receiving accommodations. Approximately 13% reported needing but not receiving accommodations. However, this percentage is only reported by females 30 years and older; there were no females aged 10–29 years who indicated facing this discrepancy (Table 4). In this sample, 38.6% of participants displayed at least one cognitive deficit among the five RBANS cognitive domains (Table 2). However, 25.9% of participants who reported that they did not need accommodations showed at least one cognitive deficit (Table 5).

**Table 4.** Accommodation Characteristics, *n* (%), by Age

Characteristics	10–19 (n = 41)	20–29 (n = 19)	30–49 (n = 59)	50–69 (n = 23)	Total (n = 142)
Have you ever needed and received accommodations?					
Needed/received	16 (39.0)	6 (31.6)	15 (25.4)	6 (26.1)	43 (30.3)
Did not need	12 (29.3)	12 (63.2)	32 (54.2)	10 (43.4)	66 (46.5)
Needed/not received	0	0	11 (18.6)	7 (30.4)	18 (12.7)
Unreported	13 (31.7)	1 (5.3)	1 (1.7)	0	15 (10.6)
Accommodation types^a^					
Environmental modifications	7 (17.1)	1 (5.3)	1 (1.7)	2 (8.7)	11 (7.7)
Extra time	8 (19.5)	5 (26.3)	3 (5.1)	1 (4.3)	17 (11.9)
Material resources	4 (9.8)	2 (10.5)	2 (3.4)	1 (4.3)	9 (6.3)
Tutoring	5 (12.2)	0	8 (13.6)	2 (8.7)	15 (10.6)
Remedial classes	3 (7.3)	1 (5.3)	5 (8.5)	1 (4.3)	10 (7.0)
Accommodation subjects^a^					
Mathematics	10 (24.4)	2 (10.5)	6 (10.2)	2 (8.7)	20 (14.1)
Speech and language	1 (2.4)	0	4 (6.8)	0	5 (3.5)

^a^ Because individuals can list more than one accommodation type or subject, accommodations-related percentages do not add up to 100%.

**Table 5.** Cognitive and Physical Descriptors in Relation to History of Academic Accommodations

Have you ever needed and received accommodations?	Needed/received (n = 27)	Did not need (n = 54)	Needed/not received (n = 18)
Height (median), feet/inches	144.78 cm	146.30 cm	146.30 cm
Cognitive deficits, *n* (%)^a^			
No deficits	12 (44.4)	40 (74.1)	10 (55.6)
One domain	10 (37.0)	9 (16.7)	7 (38.9)
Two domains	4 (14.8)	4 (7.4)	1 (5.6)
Three or more domains	1 (3.7)	1 (1.9)	0

^a^ Cognitive deficit refers to participants (*n* = 99) who provided information on accommodation history and RBANS scores. Participants who did not provide accommodation information (*n* = 2) and minors who were not administered the RBANS (*n* = 41) are not included.

Participants reported receiving extra time for tests and tasks (11.9%), material resources (6.3%), tutoring (10.6%), environmental accommodations (7.7%), and remedial classes (7.0%; Table 4).

## Discussion

This study sought to examine cognitive deficits and the procurement of academic accommodations in females with Turner syndrome. Cognitive deficits within this sample are comparable with previous reports of females with Turner syndrome, with average immediate memory, language, and attention abilities, as well as difficulties in visuospatial and delayed memory domains.^4,5,7^ Findings from this sample display progress in the obtainment of needed academic accommodations within recent decades for females with Turner syndrome. However, findings still show a disparity between cognitive deficits shown and accommodations provided to this sample (Table 5).

### Education and accommodations

Previous literature demonstrates high educational outcomes in females with Turner syndrome.^22^ In this study, ∼52% of those at or >20 years old completed postsecondary education. Although these numbers indicate a high-achieving population, this may insinuate that accommodations are unnecessary. As our findings suggest, cognitive deficits are still evident, particularly in delayed memory and visuospatial domains. Although females with Turner syndrome's outcomes undoubtably display aptitude, the use of accommodations should still be considered to foster educational attainment.

### Procurement of accommodations

Between the oldest and youngest age groups, the percentage of needed/unreceived accommodations decreased, whereas needed/received accommodations increased. Despite these progressive changes, 25.9% of this sample reported accommodations as unnecessary while displaying cognitive impairments in one or more domains (Table 5). This discrepancy may result from an unawareness that accommodations can or should be implemented, originating from students with Turner syndrome, families, or educators. Potentially, educators, learning difficulty specialists, and other professionals can inform and guide females with Turner syndrome on academic accommodations that align with their cognitive, physical, or environmental needs.

### Accommodations

Previous studies highlight the effectiveness of extended testing time in individuals with learning deficits.^23^ Among those who received accommodations, about 12% specified receiving extended testing and activity time (Table 4). This may potentially combat impaired recall ability, as evident by delayed memory deficits. Findings display that females with Turner syndrome have significantly slower task response times than typically developing controls.^10^ Extended time is a simple yet applicable accommodation for a population experiencing these deficits.

Consistent with previous literature, visuospatial impairments are evident in this sample.^11^ About 14% of accommodation-receiving participants specified having mathematics-related accommodations (*e.g.*, tutoring, remedial classes, and extended time in mathematics subjects; Table 4). Based on previous studies, small-group instruction improves students' ability to stay on task.^24^ Therefore, the individualized aspect of tutoring or small-class sizes may be advantageous to students with Turner syndrome. Furthermore, previous studies indicate that females with Turner syndrome do not display significant impairment during untimed arithmetic-based tasks.^25^ Extended activity time may be particularly relevant for visuospatial tasks, as mathematic performance is unimpaired in the absence of time constraint.^25^

Numerous accommodations, such as material resources, do not fall into one specific cognitive domain. Instead, these general accommodations may compensate for overall cognitive deficits. For example, previous studies report the effectiveness of accommodations such as noise-reduction headphones in individuals with learning deficits.^26^ General accommodations, such as headphones, may be considered to aid this population as well.

In addition, 44.4% of those who needed/received accommodations displayed no cognitive deficits. However, females with Turner syndrome may experience challenges beyond cognitive deficits, including those related to physical difficulties (*e.g.*, height, vision, and hearing). Thus, these students may benefit from environmental modifications, such as individualized classroom seating or ergonomic desk/computer workspaces. Educators and other professionals must acknowledge the need for environmental, as well as academic accommodations in students with Turner syndrome.

### Study limitations

There are limitations within this study. This sample was skewed across demographics, such as race, education level, and karyotype (Table 1). Because the majority of this sample was Caucasian, it should be noted that it is unknown whether these findings generalize to women across races and ethnicities. In addition, participants self-reported academic accommodation history, which can introduce potential self-report and recall biases. Future studies may further evaluate educational needs by employing parent/guardian validation measures or requesting educational records. In addition, 10.6% of this sample did not report or opted not to report history of academic accommodations; the majority of unreported accommodations came from the 10-19-year olds (31.7%; Table 4).

Furthermore, accommodation history interviews did not inquire about information that may affect an educational institution or individual's ability to accommodate (*e.g.*, severity of cognitive deficits, socioeconomic status, and availability of resources). This may also apply to those >60 years old, who may have attended school at a time when laws did not mandate the provision of reasonable accommodations in academic settings. This study did not inquire about accommodations needed or received at individual grade levels or subject matters. Future studies may consider evaluating associations between accommodations, grade level, and subject matter.

Finally, since the completion of this study, an updated version of the RBANS has been validated on individuals 12 years and older. Future studies may consider using this version to assess both accommodations and cognition in youth with Turner syndrome.

## Conclusions

Overall, cognitive outcomes present patterns of strengths and weaknesses in females with Turner syndrome. This population displays cognitive difficulties that may impede academic achievement. With access to these profiles, educators and health care professionals may assess educational needs of students and offer appropriate and individualized academic accommodations. Turner syndrome clinical practice guidelines underscore the need for these individuals to receive the cognitive assessments and academic accommodations necessary to compensate for learning difficulties and promote academic achievement.^27^ We emphasize these statements, recommending regular assessment of academic needs and modification of students' education plans as necessary.

With this information, educators and other health care professionals can potentially target and mitigate deficits, provide individualized classroom-based accommodations, and ultimately support educational attainment in females with Turner syndrome.

## Abbreviations Used

GED: general education development
NICHID: National Institute of Child Health and Human Development
NIH: National Institutes of Health
RBANS: Repeatable Battery for the Assessment of Neuropsychological Status
